# Adolescent motherhood and the development of adolescent Offspring: Examining the role of selection versus early environmental factors

**DOI:** 10.1016/j.ssmph.2025.101818

**Published:** 2025-05-16

**Authors:** Emla Fitzsimons, Aase Villadsen

**Affiliations:** Centre for Longitudinal Studies, University College London, 20 Bedford Way, London WC1H 0AL, United Kingdom

**Keywords:** Adolescent mothers, Adolescent outcomes, Longitudinal data, Millennium cohort study

## Abstract

**Background:**

The study examines the adolescent developmental outcomes in education, mental health, and physical health of children born to teenage mothers at the start of the millennium.

**Objective:**

It aims to understand the extent to which long-term developmental outcomes of children born to adolescent mothers are due to selection effects versus other factors.

**Methods:**

It uses longitudinal data from the UK Millennium Cohort Study. Multivariate regressions examine the extent to which the association between maternal age at birth and adolescent outcomes is explained by selection into teenage motherhood, and how the relationship is mediated by the early environment and maternal behaviours.

**Results:**

Teenage mothers are disadvantaged in terms of their backgrounds, and their children faced more adversity in their early environment. An unadjusted comparison shows that their adolescent offspring have lower academic achievement, and are more likely to be overweight or obese, but there are no differences in their socio-emotional adjustment. The ‘penalty’ from teenage motherhood in excess weight is due to negative selection into teenage motherhood. However, the differences in educational attainment of adolescents born to teenage and older mothers reflect both pre-childbearing selection and differences in the child's early environment. A decomposition analysis shows that maternal age accounts for only a low proportion of the variance in adolescent development.

**Contribution:**

The study provides the first evidence on long-term outcomes of children born to teenage mothers for the UK. It studies the entire range of key developmental outcomes. It uses a novel decomposition to examine the relative importance of different variables for explaining variation in the outcomes of interest.

## Highlights

• The study provides the first evidence on long-term outcomes of children born to teenage mothers for the UK, across key developmental outcomes.
• Adolescent offspring of teenage mothers have lower academic attainment, higher risk of excess weight, but similar socio-emotional adjustment.
• Differences in excess weight signal negative selection; education differences reflect selection and environment. Maternal age explains little of adolescent outcomes.

## 1 Introduction

As recently as two decades ago, the United Kingdom had the highest adolescent birth rate in Europe and second highest in the developed world, after the United States (Brennan, 2002). The teenage birth rate in the UK has seen a stark decrease over the period since then, with the decline in England and Wales particularly pronounced, from 29.3 live births per 1000 women aged 15–19 in 2000 to 8.4 in 2021 (Office for National Statistics, 2022), bringing the UK in line with the European Union average, which stood at 9 live births in 2020 from 14 in 2000 (The World Bank, 2022). There is evidence that this threefold reduction was mostly attributable to causes spanning several countries, including economic change, improvements in access to contraception and contraception technologies, changes in other social welfare policies and greater access of young women to education (Girma & Paton, 2015; Heap et al., 2020; Kearney & Levine, 2015; Sipsma et al., 2017). However, it also coincides with a period of increased and sustained policy focus, mainly driven by the ten-year Teenage Pregnancy Strategy, introduced in England in 1999 (Hadley et al., 2016).

The drive in policy has been underpinned by an extensive body of evidence showing that children born to adolescent mothers tend to fare out worse on various measures, including poorer health, worse cognitive and behavioural development lower schooling and lower adult earnings (Angrist & Lavy, 1996; Brooks-Gunn & Furstenberg Jr, 1986; Francesconi, 2008; Geronimus et al., 1994; López Turley, 2003; Mollborn, 2017). Much of the focus of this literature has been on early outcomes such as educational attainment, emotional and behavioural problems, and illness, accidents and injuries (Brooks-Gunn & Furstenberg Jr, 1986; Geronimus et al., 1994; Hechtman, 1989; López Turley, 2003; Mollborn, 2017).

There is less evidence on the young adult outcomes of children born to teenage mothers, with far fewer studies following the offspring to adolescence. Notable exceptions include (Jaffee et al., 2001), who sh ow worse outcomes in early adulthood in terms of early school leaving, unemployment, early parenthood, and violent offending, for a cohort born in New Zealand in the early 1970s. Shaw et al. (2006) show more disturbed psychological behaviour, poorer school performance and lower reading ability, greater likelihood of contact with the criminal justice system, and regular smoking and alcohol consumption, for a cohort born in Australia in the 1980s. More recent work by Aizer et al. (2022), which uses administrative Norwegian data covering birth born across 1967–2009, shows lower schooling, earnings and higher likelihood of teenage birth, among those born to younger adolescent mothers, aged 15–17 years old. Additionally, a Swedish study examined a range of health and health behaviours of offspring at age 19 and showed more adverse outcomes for those born to teenage mothers than mothers in their early thirties (Barclay & Myrskyla, 2016).

As teenage mothers are more likely to come from disadvantaged backgrounds, differences in offspring outcomes by maternal ages may simply reflect differences in pre-childbearing characteristics, i.e. negative selection into teenage motherhood. However, there are several other theoretical mechanisms which may explain why children born to teenage mothers do worse than those with older mothers. One factor is resources, with the lifecycle earnings hypothesis showing that teenagers are significantly more resource-constrained than their older counterparts (Featherman et al., 1988; Mirowsky & Ross, 1999), and teenage childbirth being associated with lower parental earnings and higher rates of poverty (Maynard & Garry, 1997). This may impact adversely on human capital investments such as early education and health care (Leigh & Gong, 2010). A further factor is that early parenthood may prevent teenage parents from making optimal investments in their own education and labour market participation (Duncan et al., 2018; Foster & Furstenberg Jr, 1993), further exacerbated by the fact that teenage pregnancies are more likely to be unintentional than are adult pregnancies (Wellings et al., 2013). Another contributing factor may concern the fact that adolescents tend to have lower levels of psychosocial maturity than their older counterparts (Steinberg et al., 2009), and in particular are more likely to report greater levels of stress and anxiety, with implications for their interactions with children (Guajardo et al., 2009). A recent study also considers the role of fathers, finding that fathers' underlying characteristics may also play an important role in explaining their children's worse outcomes in both the short and long run (Aizer et al., 2022).

To examine the relationship between children's human development and maternal age, we draw on data from the Millennium Cohort Study, a major UK-wide longitudinal study following participants from early life. We examine the three core domains of development in late adolescence: educational attainment, mental health and physical health. We account for a range of pre-childbearing characteristics in order to examine the extent to which differences reflect selection into teenage motherhood. We also examine the role of mediating factors, including the child's early material, emotional and educational environment, all measured using rich longitudinal data.

## 2 Methods

### 2.1 Data and sample

The Millennium Cohort Study (MCS) is a nationally representative birth cohort study that follows individuals born in the UK around the new millennium (September 2000–January 2002). A detailed description of the cohort study – including sampling frame and strategy - is provide elsewhere (Joshi & Fitzsimons, 2016). A total of 19,243 of families were recruited to the study, with the initial survey carried out at child age 9 months, followed by surveys at ages 3, 5, 7, 11, 14 and 17. For the current study we focus on first-time natural mothers and on singleton births, thereby excluding twins and triplets (1.4 % of the sample). Of the 18,948 families where the main respondent was the natural mother and the cohort child was a singleton, 7681 (41 %) were born to first-time mothers. The final sample of 4240 participants were those families who took part in the seventh survey when cohort member were age 17. The effective sample size however varies depending on the availability data for each of the age 17 outcomes (see Table 2).

### 2.2 Measures

#### 2.2.1 Maternal age at first birth

This was reported by the mother, as month and year of their own birth at the time of birth of the cohort child. The following age groups were constructed and are used in our analyses: 19 years and under, 20–24, 25–34, and 35 and above.

#### 2.2.2 Offspring outcomes at age 17

Our measures of the three major pillars of human development, including socio-emotional, cognitive and health, are described below.

*Socio-emotional development.* Emotional problems were self-reported using the Strengths and Difficulties Questionnaire (SDQ) (Goodman, 1997). Five items measure emotional problems (I get a lot of headaches, I worry a lot, I am often unhappy, I am nervous in new situations, I have many fears), with response options: ‘not true’ = 0, ‘somewhat true’ = 1, ‘certainly true = 2. The overall scale ranged from 0 to 10, and demonstrated a good interitem reliability (α = 0.74).

*Cognitive development.* Academic achievement was based on self-reported secondary school leaving results – General Certificate of Secondary Education (GCSE) results in England, Wales and Northern Ireland, and National 4 and 5 qualifications (N4 and N5) in Scotland. A binary measure of “good” academic attainment was created, defined as achieving five or more GCSEs (including maths and English) graded C or above, or five or more N5s, including maths and English, graded D or above.

*Physical development.* Excess weight was used as our measure of physical growth and development. Height and weight were measured in the home by trained interviewers. Using the British 1990 (UK90) growth reference chart for children (Freeman et al., 1995), the excess weight category included those meeting the thresholds for overweight or obese.

#### 2.2.3 Explanatory variables

An extensive set of background variables are included as correlates in estimating the association between maternal age and offspring outcomes, to help mitigate selection effects owing to omitted variables bias. These include: *Child characteristics* (gender, birthweight); *Maternal background* (ethnicity, single parent, unplanned pregnancy, maternal experience of parental separation, maternal experience of being in care as a child, education levels of maternal parents). We then adjust for a set of mediators, grouped into the following four domains: *Maternal health behaviours* (BMI pre-birth, breastfeeding, drinking during pregnancy, smoking during pregnancy, maternal physical exercise); *Educational environment* (maternal education, home learning environment, childcare arrangements, maternal word/vocabulary score); *Emotional environment* (maternal mental health, parenting type, mother-child relationship, inter-parental relationship, inter-parental abuse); *Material environment* (household income, housing tenure, social class, employment). Details of each of the explanatory variables are shown in Table S1 of the supplementary material.

### 2.3 Analyses

We first provide a description of maternal characteristics and children's early environments, along with adolescent outcomes at age 17, by maternal age at first birth. Whilst the table shows all maternal age groups, for ease of exposition we focus on comparing maternal age below 20 to maternal age 25 to 34. We focus on this comparison group to provide policy relevant results that compare teenage motherhood to a normative age group for first birth. The median age for first birth was 29 years in this UK nationally representative sample, so this guided our age range for the comparison group.

We next use multivariate regressions to examine the extent to which the association between maternal age at birth and adolescent outcomes is explained by pre-childbearing characteristics, i.e. selection into teenage motherhood. We then examine the extent to which the relationship is mediated by the child's early environment and maternal behaviours in a range of domains. Ordinary least square (OLS) regression is used for continuous outcomes and logistic regression for binary outcomes.

In order to better understand the relative contribution of maternal age to adolescent outcomes vis-a-vis other aspects of the early childhood environment, we compute a Shorrocks-Shapley decomposition of the R-squared (Shorrocks, 2013). This decomposes the overall R-squared of our regression models into the proportion explained by the different groups of variables in the model (maternal age, child characteristics, parental background, parental health behaviours, educational environment, emotional environment, material environment). We implement this using the shapley2 Stata module (Wendelspiess Chávez Juárez, 2015).

In all analyses, weights are used to adjust for the clustered sampling strategy used in the initial sweep of the MCS (Joshi & Fitzsimons, 2016). This is in combination with weights that adjust for non-random attrition between baseline and the age 17 survey, using inverse probability weights customised to this sample of first-time mothers, and which were estimated using a range of auxiliary variables predictive of attrition. Together, these weights help ensure our estimates are representative of first-time mothers in the whole of the UK (in 2000/01). All analyses are carried out using Stata version 17 (StataCorp, 2021).

### 2.4 Research Ethics

Ethical approval was obtained for all waves of the Millennium Cohort Study through the National Health Service (NHS) Research Ethics Committee (REC). Ethical approvals are listed below.

**MCS1** – 9 months (2000/1), South West MREC, MREC/01/6/19.

**MCS2** – 3 years (2003/4), London MREC, MREC/03/2/022.

**MCS3** – 5 years (2005/6), London MREC, 05/MRE02/46.

**MCS4** – 7 years (2007/8), Yorkshire MREC, 07/MRE03/32.

**MCS5** – 11 years (2011/12), Yorkshire and The Humber REC – Leeds East, 11/YH/0203.

**MCS6** – 14 years (2015/16), Central REC – London, 13/LO/1786.

**MCS7** – 17 years (2018/19), North East REC – York, 17/NE/0341.

## 3 Results

The median age at which mothers had their first child was 29 years. Those having their first child in their teens accounted for 15.3 % of the sample, 18.9 % were between 20 and 24, 55.4 % were aged 25 to 34, and 10.4 % were aged 35 or over. Of the teenage first-time mothers, the majority (88 %) were between 17 and 19 years, so very similar in age to when we observe their own children's outcomes at age 17.

### 3.1 Descriptive Statistics

#### 3.1.1 Maternal characteristics and the early environment

Table 1 displays descriptive characteristics of first-time mothers in the MCS across an extensive set of variables, providing a comparison across groups as defined by maternal age at first birth. The table includes several measures of mothers' backgrounds, alongside the educational, emotional, and material environment of the home, and maternal health behaviours. With only a few exceptions, these are all measured in the very early years of their child's life, at either 9 months or at age 3 years (variable descriptions are in Table S1 of the Supplementary Material). A general pattern is that adolescent mothers (<20) are the most disadvantaged across these domains, followed by mothers aged 20–24. Older mothers, aged 35 and above, are generally the most advantaged, followed by mothers aged 25 to 34. As noted, in the following exposition, comparisons are made between mothers aged 19 and below and mothers aged 25 to 34 (as the majority category). All comparisons highlighted in the following are statistically significant differences (p < 0.05 or below) between teen mothers and the age 25 to 34 comparison group, unless otherwise indicated, based on the confidence intervals reported in Table 1.

**Table 1:** Characteristics of mothers overall and by age at first birth.

	Total sample		Maternal age at first birth							
			35 or above		25 to 34		20 to 24		Under 20	
			N = 441		N = 2349		N = 801		N = 649	
	Sample size	% or mean	% or mean	95 % CI	% or mean	95 % CI	% or mean	95 % CI	% or mean	95 % CI
**MATERNAL BACKGROUND**										
**Mother's ethnicity**	4240									
White	3604	90.4 %	93.0 %	[90.2 %,95.0 %]	92.3 %	[90.6 %,93.8 %]	82.1 %	[77.0 %,86.2 %]	91.7 %	[87.9 %,94.4 %]
Mixed	56	1.3 %	0.6 %	[0.2 %,2.1 %]	0.8 %	[0.5 %,1.4 %]	1.8 %	[1.0 %,3.3 %]	2.8 %	[1.5 %,4.9 %]
Indian	123	2.0 %	1.5 %	[0.6 %,3.5 %]	2.0 %	[1.4 %,3.0 %]	3.3 %	[2.2 %,5.1 %]	0.5 %	[0.1 %,1.8 %]
Pakistani and Bangladeshi	250	2.9 %	0.4 %	[0.1 %,1.4 %]	1.4 %	[0.9 %,2.1 %]	9.0 %	[5.8 %,13.7 %]	2.6 %	[1.4 %,4.7 %]
Black or Black British	115	2.0 %	3.3 %	[2.0 %,5.4 %]	1.7 %	[1.1 %,2.6 %]	2.2 %	[1.2 %,4.2 %]	1.6 %	[0.7 %,3.5 %]
Other	92	1.5 %	1.2 %	[0.6 %,2.5 %]	1.7 %	[1.1 %,2.7 %]	1.5 %	[0.9 %,2.6 %]	0.8 %	[0.3 %,1.9 %]
**Single parent/carer**	4240	16.8 %	6.7 %	[4.5 %,9.7 %]	6.8 %	[5.7 %,8.2 %]	23.2 %	[19.8 %,27.0 %]	51.5 %	[46.9 %,56.2 %]
**Unplanned pregnancy**	4233	42.6 %	24.8 %	[20.6 %,29.4 %]	27.3 %	[25.2 %,29.4 %]	61.6 %	[57.4 %,65.6 %]	86.8 %	[82.9 %,89.9 %]
**Mother's parents separated**	4238	30.3 %	19.4 %	[15.9 %,23.6 %]	24.8 %	[22.8 %,26.8 %]	38.0 %	[33.6 %,42.6 %]	48.0 %	[43.0 %,53.1 %]
**Mother in care as child**	4237	1.2 %	0.1 %	[0.0 %,0.6 %]	0.8 %	[0.4 %,1.6 %]	1.3 %	[0.7 %,2.5 %]	3.5 %	[2.2 %,5.7 %]
**Mother's mother's qualifications**	2678									
Degree level or above	345	12.8 %	12.7 %	[9.1 %,17.5 %]	14.8 %	[12.9 %,16.9 %]	9.5 %	[7.0 %,12.7 %]	7.1 %	[4.0 %,12.1 %]
Other Higher Education below degree level	197	7.4 %	9.2 %	[6.5 %,12.8 %]	7.8 %	[6.1 %,9.8 %]	4.2 %	[2.7 %,6.6 %]	8.2 %	[5.0 %,13.0 %]
A levels and equivalents	196	7.0 %	7.3 %	[4.9 %,10.7 %]	6.0 %	[4.9 %,7.4 %]	7.5 %	[5.1 %,10.9 %]	11.6 %	[7.7 %,17.2 %]
GCSE/O levels and equivalents	765	32.2 %	23.7 %	[18.8 %,29.5 %]	31.1 %	[28.5 %,33.8 %]	39.3 %	[33.7 %,45.2 %]	38.0 %	[30.9 %,45.6 %]
Another type of qualification	350	12.6 %	15.0 %	[11.4 %,19.4 %]	13.6 %	[11.9 %,15.6 %]	10.2 %	[7.2 %,14.2 %]	8.0 %	[5.1 %,12.4 %]
No qualification	825	27.9 %	32.1 %	[26.7 %,38.0 %]	26.7 %	[24.0 %,29.6 %]	29.3 %	[24.1 %,35.1 %]	27.1 %	[20.6 %,34.9 %]
**Mother's father's qualifications**	2521									
Degree level or above	425	17.3 %	18.4 %	[12.8 %,25.7 %]	19.9 %	[17.0 %,23.1 %]	10.7 %	[7.8 %,14.4 %]	9.6 %	[5.5 %,16.2 %]
Other Higher Education below degree level	243	9.7 %	11.3 %	[8.0 %,15.7 %]	10.9 %	[9.2 %,12.8 %]	4.5 %	[2.8 %,7.2 %]	8.5 %	[5.0 %,14.3 %]
A levels and equivalents	209	8.4 %	8.1 %	[5.0 %,12.9 %]	7.6 %	[6.3 %,9.2 %]	10.5 %	[7.5 %,14.7 %]	10.5 %	[5.8 %,18.3 %]
GCSE/O levels and equivalents	556	24.3 %	14.6 %	[11.1 %,18.9 %]	22.7 %	[20.4 %,25.1 %]	33.2 %	[27.9 %,38.9 %]	34.8 %	[27.0 %,43.5 %]
Another type of qualification	431	16.6 %	21.8 %	[16.7 %,28.0 %]	17.6 %	[15.4 %,20.2 %]	12.4 %	[9.7 %,15.7 %]	9.4 %	[5.8 %,14.7 %]
No qualification	657	23.6 %	25.8 %	[21.2 %,31.1 %]	21.3 %	[19.0 %,23.9 %]	28.8 %	[23.5 %,34.7 %]	27.2 %	[20.9 %,34.6 %]
**EDUCATIONAL ENVIRONMENT**										
**Mother's own qualifications**	4239									
NVQ level 1	277	7.7 %	3.2 %	[1.7 %,6.2 %]	4.3 %	[3.4 %,5.5 %]	9.5 %	[7.3 %,12.4 %]	20.5 %	[16.4 %,25.4 %]
NVQ level 2	1119	28.6 %	20.4 %	[15.6 %,26.2 %]	25.3 %	[22.8 %,27.9 %]	34.1 %	[30.1 %,38.3 %]	39.4 %	[34.8 %,44.2 %]
NVQ level 3	715	16.2 %	13.2 %	[9.8 %,17.6 %]	15.0 %	[13.3 %,16.8 %]	25.3 %	[22.1 %,28.8 %]	11.5 %	[8.6 %,15.2 %]
NVQ level 4	1416	32.2 %	45.2 %	[39.1 %,51.4 %]	44.3 %	[41.3 %,47.3 %]	14.0 %	[11.6 %,16.8 %]	2.1 %	[1.1 %,3.8 %]
NVQ level 5	213	4.5 %	11.5 %	[8.1 %,16.0 %]	5.7 %	[4.7 %,7.0 %]	0.6 %	[0.2 %,1.5 %]	0.0 %	
Overseas qual only	114	2.1 %	2.8 %	[1.5 %,5.2 %]	1.8 %	[1.3 %,2.5 %]	3.4 %	[2.3 %,4.9 %]	1.0 %	[0.4 %,2.3 %]
No qualifications	385	8.8 %	3.7 %	[2.1 %,6.4 %]	3.6 %	[2.7 %,4.8 %]	13.2 %	[10.3 %,16.8 %]	25.5 %	[21.2 %,30.4 %]
**Home learning environment**	3173	12.13	11.69	[11.10,12.27]	12.39	[12.08,12.71]	11.74	[11.24,12.23]	11.86	[11.33,12.38]
**Maternal word score**	3760	11.63	14.19	[13.59,14.79]	12.49	[12.23,12.76]	9.48	[9.18,9.78]	8.68	[8.27,9.09]
**Childcare**	3862									
Main parent or partner	2213	59.3 %	51.0 %	[45.9 %,56.0 %]	51.5 %	[48.9 %,54.0 %]	70.9 %	[66.5 %,75.0 %]	82.9 %	[78.0 %,86.9 %]
Relative or friend	930	21.8 %	15.9 %	[12.5 %,20.2 %]	24.8 %	[22.0 %,27.7 %]	23.5 %	[19.8 %,27.7 %]	12.2 %	[9.0 %,16.4 %]
Childminder or nursery	719	18.9 %	33.1 %	[28.9 %,37.5 %]	23.8 %	[21.6 %,26.1 %]	5.5 %	[4.1 %,7.4 %]	4.8 %	[3.0 %,7.7 %]
**EMOTIONAL ENVIRONMENT**										
**Maternal psychological distress**	4124	1.52	1.43	[1.28,1.58]	1.33	[1.25,1.40]	1.78	[1.63,1.93]	1.98	[1.80,2.17]
**Mother-child relationship**	3498	63.76	64.85	[64.20,65.51]	64.45	[64.13,64.77]	62.47	[61.79,63.14]	61.59	[60.73,62.45]
**Inter-parental relationship**	2848	15.29	15.07	[14.60,15.54]	15.60	[15.41,15.79]	14.65	[14.25,15.05]	14.30	[13.67,14.93]
**Parenting type**	3871									
Permissive	908	22.0 %	22.3 %	[17.9 %,27.5 %]	19.7 %	[17.5 %,22.0 %]	24.8 %	[21.2 %,28.7 %]	27.7 %	[22.9 %,33.0 %]
Authoritarian	441	11.1 %	10.6 %	[7.4 %,14.9 %]	8.9 %	[7.3 %,10.8 %]	13.6 %	[11.0 %,16.7 %]	17.2 %	[13.6 %,21.5 %]
Neglectful	227	5.5 %	3.7 %	[2.1 %,6.5 %]	3.1 %	[2.4 %,4.1 %]	8.0 %	[6.0 %,10.6 %]	13.4 %	[10.0 %,17.9 %]
Authoritative	2295	61.4 %	63.4 %	[57.4 %,69.0 %]	68.3 %	[65.4 %,71.1 %]	53.6 %	[48.2 %,58.9 %]	41.7 %	[36.6 %,46.9 %]
**Inter-parental abuse**	3627									
No	2595	71.0 %	83.1 %	[78.9 %,86.7 %]	82.5 %	[80.4 %,84.4 %]	59.4 %	[54.7 %,63.9 %]	32.0 %	[27.5 %,37.0 %]
Yes	342	10.0 %	9.6 %	[6.9 %,13.1 %]	10.0 %	[8.6 %,11.5 %]	12.7 %	[10.0 %,16.0 %]	7.4 %	[5.2 %,10.4 %]
No partner	690	19.0 %	7.3 %	[5.0 %,10.6 %]	7.6 %	[6.3 %,9.1 %]	27.9 %	[23.8 %,32.4 %]	60.6 %	[55.5 %,65.5 %]
**MATERIAL ENVIRONMENT**										
**Household income (weekly)**	4174	£356	£496	[£472,$521]	£432	[£416,£447]	£232	[£220,£244]	£149	[£140,£158]
**Housing tenure**	4240									
Own outright	141	2.8 %	5.5 %	[3.6 %,8.4 %]	3.0 %	[2.2 %,3.9 %]	2.8 %	[1.8 %,4.2 %]	0.3 %	[0.1 %,0.6 %]
Mortgage	2566	60.7 %	81.1 %	[76.5 %,85.0 %]	79.7 %	[77.4 %,81.9 %]	34.7 %	[30.1 %,39.6 %]	10.1 %	[7.5 %,13.4 %]
Rent public	743	18.1 %	4.9 %	[3.2 %,7.4 %]	6.7 %	[5.4 %,8.3 %]	30.4 %	[25.9 %,35.3 %]	53.2 %	[47.2 %,59.0 %]
Rent private	386	9.4 %	5.5 %	[3.6 %,8.3 %]	6.8 %	[5.6 %,8.3 %]	15.0 %	[11.9 %,18.7 %]	14.4 %	[10.8 %,18.9 %]
Other	404	9.0 %	3.0 %	[1.5 %,5.9 %]	3.8 %	[3.0 %,4.8 %]	17.2 %	[13.9 %,21.1 %]	22.1 %	[18.0 %,26.8 %]
**Highest social class in household**	4240									
Managerial and professional	2090	50.7 %	72.5 %	[67.2 %,77.2 %]	67.7 %	[64.7 %,70.7 %]	23.8 %	[20.2 %,27.8 %]	7.6 %	[5.2 %,11.0 %]
Intermediate	568	13.5 %	9.1 %	[6.5 %,12.7 %]	13.7 %	[12.0 %,15.6 %]	16.9 %	[14.2 %,19.9 %]	11.5 %	[8.7 %,15.0 %]
Small employer and self-employed	183	4.1 %	4.8 %	[3.1 %,7.5 %]	3.8 %	[2.9 %,5.1 %]	5.0 %	[3.6 %,6.9 %]	3.8 %	[2.4 %,6.0 %]
Lower supervisory and technical	354	8.2 %	3.2 %	[1.8 %,5.6 %]	5.8 %	[4.8 %,7.0 %]	15.2 %	[12.1 %,18.9 %]	12.0 %	[9.3 %,15.3 %]
Semi-routine and routine	859	19.8 %	7.2 %	[4.8 %,10.7 %]	7.9 %	[6.6 %,9.5 %]	35.5 %	[31.4 %,39.9 %]	51.8 %	[47.3 %,56.3 %]
Unclassified	186	3.6 %	3.1 %	[1.6 %,6.2 %]	1.0 %	[0.6 %,1.6 %]	3.7 %	[2.3 %,5.7 %]	13.2 %	[10.1 %,17.2 %]
**Combined labour market status in household**	4240									
Both in work	2232	51.6 %	66.1 %	[61.5 %,70.5 %]	64.0 %	[61.6 %,66.2 %]	36.3 %	[32.7 %,40.2 %]	15.7 %	[12.6 %,19.5 %]
Mother in work, partner not	94	2.3 %	3.0 %	[1.7 %,5.2 %]	2.4 %	[1.7 %,3.3 %]	2.7 %	[1.7 %,4.4 %]	1.1 %	[0.5 %,2.6 %]
Partner in work, mother not	1013	25.0 %	22.9 %	[19.1 %,27.2 %]	24.9 %	[22.8 %,27.1 %]	30.7 %	[27.2 %,34.5 %]	19.9 %	[15.8 %,24.6 %]
Both not in work	211	4.3 %	1.3 %	[0.5 %,3.7 %]	1.9 %	[1.4 %,2.7 %]	7.0 %	[5.1 %,9.5 %]	11.7 %	[9.2 %,14.8 %]
Mother in work or on leave, no partner	243	5.6 %	5.2 %	[3.2 %,8.2 %]	3.6 %	[2.8 %,4.7 %]	8.5 %	[6.7 %,10.8 %]	9.5 %	[7.2 %,12.6 %]
Mother not in work nor on leave, no partner	447	11.1 %	1.5 %	[0.6 %,3.5 %]	3.2 %	[2.5 %,4.1 %]	14.7 %	[11.8 %,18.2 %]	42.0 %	[37.6 %,46.6 %]
**MATERNAL HEALTH BEHAVIOURS**										
**Pre-birth BMI**	4240	23.35	24.00	[23.47,24.54]	23.82	[23.60,24.04]	23.09	[22.71,23.47]	21.44	[21.09,21.80]
**Breastfeed child**	3947	76.5 %	89.6 %	[85.7 %,92.5 %]	84.5 %	[82.5 %,86.4 %]	69.5 %	[65.2 %,73.5 %]	47.3 %	[41.9 %,52.7 %]
**Drinking alcohol during pregnancy**	4240									
Never	2942	66.6 %	56.3 %	[51.0 %,61.4 %]	62.1 %	[59.6 %,64.7 %]	75.9 %	[71.7 %,79.7 %]	77.9 %	[73.5 %,81.8 %]
Less than once a month	620	16.1 %	19.3 %	[15.4 %,24.0 %]	18.0 %	[16.1 %,20.0 %]	13.4 %	[10.5 %,17.0 %]	10.4 %	[8.0 %,13.6 %]
1-2 times a month	311	8.0 %	9.4 %	[6.7 %,12.9 %]	9.7 %	[8.4 %,11.2 %]	5.5 %	[3.9 %,7.7 %]	3.8 %	[2.2 %,6.3 %]
1-2 times a week	292	7.5 %	11.5 %	[8.6 %,15.3 %]	8.4 %	[7.1 %,10.0 %]	4.1 %	[2.8 %,5.9 %]	5.4 %	[3.3 %,8.6 %]
3-4 times a week or more	75	1.9 %	3.5 %	[2.1 %,6.0 %]	1.7 %	[1.3 %,2.4 %]	1.1 %	[0.4 %,2.4 %]	2.5 %	[1.3 %,4.5 %]
**Smoking during pregnancy**	4240									
Never smoked	2381	52.4 %	63.4 %	[58.0 %,68.5 %]	59.7 %	[57.4 %,62.1 %]	44.4 %	[40.3 %,48.6 %]	28.0 %	[23.6 %,32.9 %]
Ex-smoker before pregnancy	668	16.9 %	21.0 %	[17.2 %,25.4 %]	18.8 %	[17.0 %,20.8 %]	14.1 %	[11.2 %,17.6 %]	10.9 %	[8.2 %,14.3 %]
Current smoker but not during pregnancy	622	16.0 %	9.3 %	[6.3 %,13.4 %]	12.9 %	[11.4 %,14.6 %]	20.5 %	[17.5 %,24.0 %]	25.9 %	[21.4 %,31.0 %]
Smoked during pregnancy	569	14.7 %	6.3 %	[4.2 %,9.3 %]	8.5 %	[7.3 %,10.0 %]	21.0 %	[17.2 %,25.3 %]	35.2 %	[30.7 %,39.9 %]
**Mother's physical exercise**	3978									
Never	1011	24.1 %	19.0 %	[15.2 %,23.4 %]	19.5 %	[17.2 %,22.0 %]	28.5 %	[24.8 %,32.5 %]	40.4 %	[35.5 %,45.6 %]
At least one a year to every few months	405	10.4 %	9.7 %	[6.7 %,13.7 %]	11.7 %	[10.1 %,13.5 %]	8.0 %	[6.0 %,10.5 %]	9.1 %	[6.7 %,12.3 %]
At least once a month	443	11.2 %	10.9 %	[8.2 %,14.4 %]	10.3 %	[8.9 %,11.9 %]	13.2 %	[10.7 %,16.1 %]	12.7 %	[9.8 %,16.2 %]
Once or twice a week	1020	25.4 %	26.5 %	[22.0 %,31.5 %]	27.8 %	[25.6 %,30.2 %]	22.5 %	[19.4 %,26.0 %]	18.5 %	[15.3 %,22.3 %]
Every day or almost every day	1099	28.9 %	34.0 %	[29.1 %,39.2 %]	30.7 %	[28.3 %,33.3 %]	27.9 %	[24.0 %,32.0 %]	19.3 %	[15.4 %,23.8 %]

**Table 2:** Offspring characteristics and outcomes at age 17 overall and by maternal age at first birth.

	Total sample			Maternal age at first birth							
				35 or above		25 to 34		20 to 24		Under 20	
				N = 441		N = 2349		N = 801		N = 649	
	Sample size	% or mean		% or mean	CI	% or mean	CI	% or mean	CI	% or mean	CI
**CHARACTERISTICS**											
**Sex at birth**	4240										
Female	2104	48.5 %		51.2 %	[46.0 %,56.3 %]	47.8 %	[45.3 %,50.3 %]	48.4 %	[44.5 %,52.2 %]	49.0 %	[43.9 %,54.2 %]
Male	2136	51.5 %		48.8 %	[43.7 %,54.0 %]	52.2 %	[49.7 %,54.7 %]	51.6 %	[47.8 %,55.5 %]	51.0 %	[45.8 %,56.1 %]
**Ethnicity**	4240										
White	3549	89.2 %		91.2 %	[87.9 %,93.6 %]	91.5 %	[89.6 %,93.2 %]	80.3 %	[75.1 %,84.6 %]	90.2 %	[86.2 %,93.1 %]
Mixed	139	3.3 %		4.6 %	[2.8 %,7.5 %]	2.6 %	[2.0 %,3.4 %]	4.1 %	[2.7 %,6.2 %]	4.1 %	[2.5 %,6.6 %]
Indian	120	1.8 %		1.5 %	[0.6 %,3.5 %]	1.6 %	[1.0 %,2.5 %]	3.4 %	[2.2 %,5.2 %]	0.7 %	[0.3 %,2.1 %]
Pakistani and Bangladeshi	250	2.8 %		0.2 %	[0.0 %,1.4 %]	1.3 %	[0.9 %,2.0 %]	8.8 %	[5.6 %,13.5 %]	2.5 %	[1.3 %,4.6 %]
Black or Black British	114	1.9 %		2.0 %	[1.1 %,3.8 %]	1.8 %	[1.2 %,2.7 %]	2.3 %	[1.2 %,4.2 %]	1.8 %	[0.9 %,3.9 %]
Other	68	1.0 %		0.5 %	[0.2 %,1.0 %]	1.2 %	[0.7 %,1.9 %]	1.1 %	[0.6 %,2.1 %]	0.7 %	[0.3 %,1.7 %]
**Country**	4240										
England	2738	83.1 %		85.2 %	[81.7 %,88.1 %]	84.0 %	[81.6 %,86.1 %]	81.1 %	[77.4 %,84.3 %]	81.2 %	[77.4 %,84.5 %]
Wales	612	5.1 %		3.6 %	[2.4 %,5.5 %]	4.7 %	[3.6 %,6.1 %]	6.0 %	[4.6 %,7.9 %]	6.3 %	[4.8 %,8.1 %]
Scotland	512	8.7 %		9.3 %	[7.2 %,11.9 %]	8.4 %	[6.9 %,10.1 %]	8.8 %	[6.6 %,11.5 %]	9.2 %	[6.9 %,12.2 %]
Northern Ireland	378	3.1 %		1.9 %	[1.2 %,2.9 %]	3.0 %	[2.5 %,3.6 %]	4.1 %	[2.9 %,5.9 %]	3.3 %	[2.5 %,4.5 %]
**AGE 17 OUTCOMES**											
**Academic attainment**											
5 or more C-A star GCSEs	3948	69.8 %	SD = 45.9	81.7 %	[77.1 %,85.6 %]	78.1 %	[75.7 %,80.3 %]	58.6 %	[53.9 %,63.1 %]	42.8 %	[37.4 %,48.5 %]
**Mental Health**											
SDQ emotional symptoms (z-scores)	3918	3.51	SD = 2.49	3.66	[3.43,3.89]	3.41	[3.30,3.51]	3.64	[3.46,3.81	3.68	[3.45,3.90]
**Physical health**											
Excess weight (overweight or obese)	3756	34.6 %	SD = 47.6	28.5 %	[23.2 %,34.4 %]	32.5 %	[30.0 %,35.0 %]	40.9 %	[36.6 %,45.3 %]	40.3 %	[35.8 %,44.9 %]

*Maternal background***.**Table 1 shows that teenage mothers are less likely than mothers aged 25–34 to come from homes where their mother had a degree (7 % v 15 %); more likely to have experienced their parents separate (48 % versus 25 %), and more likely to have spent time in care as a child (3.5 % versus 0.8 %). The majority of teenage pregnancies (87 %) were unplanned, compared to 27 % of the comparison group, and over half of teenage mothers (52 %) were single parents when their child was 9 months old, compared to just 7 % of mothers aged 25 to 34.

*Educational environment.* A quarter (26 %) of teenage mothers had no educational qualifications versus 4 % of mothers aged 25 to 34, teenage mothers had a lower vocabulary score (8.7 vs 14.5), and only 5 % used formal childcare versus 24 % of mothers in the comparison group. There was no discernible difference in the home learning environment, with young mothers’ scores on this measure not significantly different to those aged 25 to 34 (11.9 vs 12.4).

*Emotional environment.* Teenage mothers reported higher levels of psychological distress (2.0 vs 1.3), a lower quality relationship with both their child and their partner, and they were less likely to display an authoritative parenting style (42 % vs 68 %). Although a higher proportion of teenage mothers in a relationship experienced inter-parental abuse (18.7 % vs 10.8 %), overall because a much higher proportion were single, they were no more likely to bring up their child in a household with abuse, 7.4 % versus 10.0 % for mothers aged 25 to 24, and not a statistically significant difference.

*Material environment.* Household weekly income was nearly threefold lower for teenage mothers (£149) compared to mothers aged 25–34 (£432), and far fewer teenage mothers had professional or managerial jobs (8 % vs 68 %). Only 16 % were in households with two earners, compared to 64 % of mothers aged 25–34. 42 % of teenage mothers were not in work and had no partner, compared to just 3 % of the older mothers in the comparison group.

*Maternal health behaviours.* A mixed picture emerged regarding health behaviours. Teenage mothers reported a significantly lower pre-birth BMI than mothers aged 25 to 34; noting that BMI is generally increasing with age in the population (NHS Digital, 2020). However, adolescent mothers reported less frequency of exercise than the older comparison group. Rates of ever having breastfed were much lower in teenage mothers (47 % vs 85 %). Teenage mothers also reported higher rates of smoking during pregnancy (35 % vs 9 %), however they were also more likely to abstain from drinking alcohol during pregnancy (78 % vs 62 %).

Our main analyses focus on maternal characteristics and child environments measured in the initial sweeps of the MCS, however we note from additional analyses (not shown) that mothers having their first child in their teens continue to experience disadvantage throughout their children's childhood.

#### 3.1.2 Child characteristics and outcomes at age 17

Turning to the children of first-time mothers in the overall sample (Table 2), around half (52 %) were male, 89 % were of White ethnic origin, and around 83 % were residing in England at the time of the baseline survey.

Looking at children's outcomes in late adolescence, at age 17, Table 2 shows differences across maternal age groups. Compared to those born to mothers aged 25 to 34, children of teenage mothers were significantly less likely to achieve ‘good’ GCSE results (43 % vs 78 %). Looking at mental health, we observe that whilst offspring of teenage mothers had a higher score on SDQ emotional problems, differences relative to the children of mothers aged 25 to 34 were small and not statistically significant. Turning to physical development, adolescents born to teenage mothers were seven percentage points more likely to be overweight or obese (40 % vs 33 %), which is a statistically significant difference.

### 3.2 Regression estimates

#### 3.2.1 Adolescent outcomes

In this section we present estimates of the association between maternal age and adolescent development. We first show the ‘raw’ association (Model 1), and then examine the extent to which it reflects selection into teenage motherhood (Model 2), i.e. the fact that teenage mothers tend to be more disadvantaged than older mothers aged 25 to 34 prior to childbearing, as Table 1 earlier on clearly displayed. We also show fully adjusted models for each outcome, i.e. controlling for aspects of the environment on the causal pathway between teenage motherhood and offspring adolescent development (Model 3). The full regression estimates for each outcome are shown in Table 3, Table 4, Table 5, for mental health, educational attainment and physical development respectively.

**Table 3:** Regression estimates: mental health (SDQ emotional problems).

	Model 1 Unadjusted	se	Model 2 Adjusted exogenous	se	Model 3 Adjusted exogenous and mediators	se
	Std coef		Std coef		Std coef	
**MATERNAL AGE**						
**Maternal age at first birth** (ref. 25–34)						
35 or over	0.10	(0.07)	0.08	(0.06)	0.09+	(0.06)
20 to 24	0.09+	(0.05)	0.10+	(0.05)	0.03	(0.05)
Under 20	0.11+	(0.06)	0.07	(0.06)	−0.02	(0.07)
**CHILD CHARACTERISTICS**						
**Male**			−0.62∗∗∗	(0.04)	−0.62∗∗∗	(0.04)
**Birth weight in kg**			−0.12∗∗∗	(0.03)	−0.11∗∗∗	(0.03)
**MATERNAL BACKGROUND**						
**Ethnicity (ref White)**						
Mixed			−0.20	(0.18)	−0.23	(0.17)
Indian			−0.27∗	(0.12)	−0.19+	(0.10)
Pakistani and Bangladeshi			−0.35∗∗∗	(0.08)	−0.35∗∗∗	(0.09)
Black or Black British			−0.22+	(0.13)	−0.25+	(0.14)
Other			−0.12	(0.14)	−0.09	(0.13)
**Planned pregnancy**			0.04	(0.04)	0.08∗	(0.04)
**Mother's parents separated**			0.09∗	(0.04)	0.06	(0.04)
**Mother in care as child**			0.27+	(0.15)	0.18	(0.15)
**Mother's mother's qualifications** (ref. No qualifications)						
Degree level or above			−0.07	(0.08)	−0.08	(0.08)
Other Higher Education below degree level			−0.09	(0.08)	−0.07	(0.08)
A levels and equivalents			0.04	(0.10)	0.04	(0.10)
GCSE/O levels and equivalents			0.05	(0.07)	0.07	(0.07)
Another type of qualification			0.09	(0.08)	0.10	(0.08)
**Mother's father's qualifications** (ref. No qualifications)						
Degree level or above			−0.06	(0.07)	−0.05	(0.07)
Other Higher Education below degree level			0.21∗	(0.09)	0.17∗	(0.08)
A levels and equivalents			0.03	(0.09)	0.02	(0.08)
GCSE/O levels and equivalents			0.05	(0.08)	0.05	(0.07)
Another type of qualification			−0.09	(0.07)	−0.09	(0.07)
**EDUCATIONAL ENVIRONMENT**						
**Mother's own qualifications** (ref. No qualifications)						
NVQ level 1					−0.05	(0.09)
NVQ level 2					0.08	(0.07)
NVQ level 3					0.12	(0.08)
NVQ level 4					0.06	(0.08)
NVQ level 5					−0.06	(0.11)
Overseas qual only					0.13	(0.12)
**Home learning environment**					0.02	(0.02)
**Childcare** (ref. mother or partner)						
Relative or friend					−0.09	(0.06)
Childminder or nursery					−0.03	(0.05)
**Maternal word score**					0.07∗∗∗	(0.02)
**EMOTIONAL ENVIRONMENT**						
**Maternal psychological distress**					0.06∗∗	(0.02)
**Single parent/carer**					0.06	(0.10)
**Parenting type**						
Permissive					−0.07	(0.05)
Authoritarian					−0.12+	(0.07)
Neglectful					0.21∗	(0.09)
**Mother-child relationship**					−0.05∗∗	(0.02)
**Inter-parental relationship**					−0.03	(0.02)
**Inter-parental abuse**					−0.06	(0.06)
**MATERIAL ENVIRONMENT**						
**Household income (weekly)**					−0.02∗	(0.01)
**Housing tenure (ref. mortgage)**						
Own outright					−0.13	(0.09)
Rent public					−0.03	(0.06)
Rent private					0.04	(0.07)
Other					−0.03	(0.08)
**Highest social class in household** (Ref. Managerial and Professional)						
Intermediate					−0.01	(0.06)
Small employer and self-employed					−0.21∗∗	(0.08)
Lower supervisory and technical					0.02	(0.08)
Semi-routine and routine					0.11	(0.07)
Unclassifiable					0.01	(0.14)
**Combined labour market status in household** (Ref. Both in work)						
Mother in work, partner not					−0.00	(0.12)
Partner in work, mother not					0.03	(0.05)
Both not in work					−0.05	(0.10)
Mother in work or on leave, no partner					−0.05	(0.10)
**MATERNAL HEALTH BEHAVIOURS**						
**Pre-birth BMI**					0.01+	(0.00)
**Breastfeed child**					0.05	(0.05)
**Drinking during pregnancy** (ref. Never)						
Less than once a month					−0.05	(0.05)
1-2 times a month					−0.14∗	(0.07)
1-2 times a week					−0.06	(0.06)
3-4 times a week or more					−0.19	(0.11)
**Smoking during pregnancy** (ref. Never smoked)						
Ex-smoker before pregnancy					0.05	(0.05)
Current smoker but not during pregnancy					0.12+	(0.06)
Smoked during pregnancy					0.05	(0.06)
**Mother's physical exercise** (ref. Never)						
At least one a year to every few months						(0.06)
At least once a month					−0.05	(0.07)
Once or twice a week					−0.15∗∗	(0.05)
Every day or almost every day					−0.12∗	(0.05)

Observations	3918		3918		3918	
R-squared	0.002		0.127		0.154	

Notes: Dummy indicators (not shown here) are used where information on predictor variables is missing.

∗∗∗p < 0.001, ∗∗p < 0.01, ∗p < 0.05, + p < 0.10.

Mean SDQ emotional symptoms is 3.52 (SD = 2.49). Note that standardized measure (z-score) is used in analyses.

**Table 4:** Regression estimates: excess weight (overweight or obese).

	Model 1 Unadjusted	se	Model 2 Adjusted exogenous	se	Model 3 Adjusted exogenous and mediators	se
	Odds Ratio		Odds Ratio		Odds Ratio	
**MATERNAL AGE**						
**Maternal age at first birth** (ref. 25–34)						
35 or over	0.83	(0.12)	0.86	(0.12)	0.92	(0.15)
20 to 24	1.44∗∗∗	(0.16)	1.25+	(0.15)	1.00	(0.14)
Under 20	1.40∗∗	(0.17)	1.14	(0.16)	0.89	(0.15)
**CHILD CHARACTERISTICS**			0.95	(0.08)		
**Male**			1.13	(0.08)	0.93	(0.09)
**Birth weight in kg**					1.13	(0.09)
**MATERNAL BACKGROUND**						
**Ethnicity (ref White)**						
Mixed			0.57	(0.22)	0.48	(0.22)
Indian			1.29	(0.29)	1.75∗	(0.41)
Pakistani and Bangladeshi			1.11	(0.22)	1.52+	(0.36)
Black or Black British			1.65∗	(0.36)	1.28	(0.33)
Other			1.19	(0.26)	1.38	(0.38)
**Planned pregnancy**			0.79∗	(0.08)	0.87	(0.09)
**Mother's parents separated**			1.09	(0.10)	1.02	(0.10)
**Mother in care as child**			1.86	(0.84)	1.45	(0.57)
**Mother's mother's qualifications** (ref. No qualifications)						
Degree level or above			0.87	(0.16)	1.03	(0.21)
Other Higher Education below degree level			0.57∗	(0.13)	0.72	(0.17)
A levels and equivalents			1.00	(0.21)	1.19	(0.25)
GCSE/O levels and equivalents			0.95	(0.13)	1.06	(0.17)
Another type of qualification			0.71+	(0.15)	0.81	(0.17)
**Mother's father's qualifications** (ref. No qualifications)						
Degree level or above			1.22	(0.24)	1.45+	(0.29)
Other Higher Education below degree level			0.90	(0.20)	0.95	(0.23)
A levels and equivalents			0.92	(0.19)	1.02	(0.22)
GCSE/O levels and equivalents			1.03	(0.19)	1.15	(0.22)
Another type of qualification			1.13	(0.22)	1.25	(0.24)
**EDUCATIONAL ENVIRONMENT**						
**Mother's own qualifications** (ref. No qualifications)						
NVQ level 1					1.27	(0.31)
NVQ level 2					1.20	(0.26)
NVQ level 3					1.15	(0.25)
NVQ level 4					1.26	(0.27)
NVQ level 5					1.47	(0.40)
Overseas qual only					1.23	(0.35)
**Home learning environment**					1.12∗	(0.05)
**Childcare** (ref. mother or partner)						
Relative or friend					1.11	(0.14)
Childminder or nursery					0.92	(0.12)
**Maternal word score**					0.85∗∗	(0.05)
**EMOTIONAL ENVIRONMENT**						
**Maternal psychological distress**					1.05	(0.05)
**Single parent/carer**					1.24	(0.24)
**Parenting type**						
Permissive					1.00	(0.11)
Authoritarian					0.94	(0.15)
Neglectful					1.23	(0.26)
**Mother-child relationship**					1.07	(0.06)
**Inter-parental relationship**					0.94	(0.05)
**Inter-parental abuse**					0.94	(0.14)
**MATERIAL ENVIRONMENT**						
**Household income (weekly)**					1.04	(0.03)
**Housing tenure (ref. mortgage)**						
Own outright					0.67	(0.17)
Rent public					1.44∗	(0.20)
Rent private					1.29+	(0.20)
Other					1.18	(0.21)
**Highest social class in household** (Ref. Managerial and Professional)						
Intermediate					0.78+	(0.11)
Small employer and self-employed					0.93	(0.20)
Lower supervisory and technical					1.59∗∗	(0.27)
Semi-routine and routine					1.35∗	(0.20)
Unclassifiable					1.01	(0.26)
**Combined labour market status in household** (Ref. Both in work)						
Mother in work, partner not					0.95	(0.29)
Partner in work, mother not					0.94	(0.11)
Both not in work					1.27	(0.33)
Mother in work or on leave, no partner					0.71	(0.18)
**MATERNAL HEALTH BEHAVIOURS**						
**Pre-birth BMI**					1.14∗∗∗	(0.01)
**Breastfeed child**					0.88	(0.10)
**Drinking during pregnancy** (ref. Never)						
Less than once a month					0.91	(0.11)
1-2 times a month					0.87	(0.15)
1-2 times a week					0.93	(0.16)
3-4 times a week or more					0.75	(0.22)
**Smoking during pregnancy** (ref. Never smoked)						
Ex-smoker before pregnancy					1.02	(0.14)
Current smoker but not during pregnancy					1.12	(0.15)
Smoked during pregnancy					1.33+	(0.20)
**Mother's physical exercise** (ref. Never)						
At least one a year to every few months					0.88	(0.16)
At least once a month					1.16	(0.15)
Once or twice a week					0.86	(0.11)
Every day or almost every day					1.05	(0.13)

Observations	3756		3756		3756	
Pseudo R-squared	0.006		0.015		0.077	

Notes: Dummy indicators (not shown here) are used where information on predictor variables is missing.

∗∗∗p < 0.001, ∗∗p < 0.01, ∗p < 0.05, + p < 0.10.

Mean excess weight is 34.6 % (SD = 47.6).

**Table 5:** Regression estimates: academic achievement (GCSE results graded A∗ to C).

	Model 1 Unadjusted	se	Model 2 Adjusted exogenous	se	Model 3 Adjusted exogenous and mediators	se
	Odds Ratio		Odds Ratio		Odds Ratio	
**MATERNAL AGE**						
**Maternal age at first birth** (ref. 25–34)						
35 or over	1.26	(0.18)	1.12	(0.17)	0.93	(0.15)
20 to 24	0.40∗∗∗	(0.05)	0.52∗∗∗	(0.06)	1.08	(0.18)
Under 20	0.21∗∗∗	(0.03)	0.34∗∗∗	(0.05)	1.02	(0.19)
**CHILD CHARACTERISTICS**						
**Male**			0.61∗∗∗	(0.05)	0.59∗∗∗	(0.05)
**Birth weight in kg**			1.30∗∗	(0.11)	1.21∗	(0.11)
**MATERNAL BACKGROUND**						
**Ethnicity (ref White)**						
Mixed			1.04	(0.41)	1.37	(0.51)
Indian			2.96∗∗	(1.13)	2.95∗∗	(1.18)
Pakistani and Bangladeshi			1.27	(0.23)	1.61∗	(0.37)
Black or Black British			0.77	(0.22)	1.45	(0.46)
Other			1.77∗	(0.49)	2.95∗∗∗	(0.84)
**Planned pregnancy**			1.31∗∗	(0.13)	1.07	(0.12)
**Mother's parents separated**			0.65∗∗∗	(0.07)	0.74∗∗	(0.08)
**Mother in care as child**			0.70	(0.25)	1.23	(0.42)
**Mother's mother's qualifications** (ref. No qualifications)						
Degree level or above			1.37	(0.28)	1.02	(0.24)
Other Higher Education below degree level			1.62+	(0.42)	1.15	(0.32)
A levels and equivalents			0.74	(0.17)	0.58∗	(0.14)
GCSE/O levels and equivalents			1.13	(0.19)	1.00	(0.19)
Another type of qualification			1.42	(0.33)	1.23	(0.32)
**Mother's father's qualifications** (ref. No qualifications)						
Degree level or above			1.64∗	(0.33)	1.25	(0.30)
Other Higher Education below degree level			0.99	(0.25)	0.81	(0.22)
A levels and equivalents			1.13	(0.27)	0.90	(0.23)
GCSE/O levels and equivalents			1.26	(0.26)	1.22	(0.26)
Another type of qualification			1.30	(0.24)	1.18	(0.24)
**EDUCATIONAL ENVIRONMENT**						
**Mother's own qualifications** (ref. No qualifications)						
NVQ level 1					0.95	(0.19)
NVQ level 2					1.27	(0.21)
NVQ level 3					1.23	(0.24)
NVQ level 4					1.50∗	(0.29)
NVQ level 5					1.27	(0.39)
Overseas qual only					1.66+	(0.47)
**Home learning environment**					1.10∗	(0.06)
**Childcare** (ref. mother or partner)						
Relative or friend					1.14	(0.18)
Childminder or nursery					1.05	(0.18)
**Maternal word score**					1.29∗∗∗	(0.08)
**EMOTIONAL ENVIRONMENT**						
**Maternal psychological distress**					1.03	(0.05)
**Single parent/carer**					0.90	(0.21)
**Parenting type**						
Permissive					0.82+	(0.09)
Authoritarian					0.94	(0.16)
Neglectful					0.56∗∗	(0.12)
**Mother-child relationship**					1.03	(0.06)
**Inter-parental relationship**					1.12+	(0.07)
**Inter-parental abuse**					0.94	(0.17)
**MATERIAL ENVIRONMENT**						
**Household income (weekly)**					1.17∗∗∗	(0.04)
**Housing tenure (ref. mortgage)**						
Own outright					1.19	(0.37)
Rent public					0.60∗∗∗	(0.09)
Rent private					0.67∗	(0.12)
Other					1.12	(0.21)
**Highest social class in household** (Ref. Managerial and Professional)						
Intermediate					0.84	(0.13)
Small employer and self-employed					0.57∗∗	(0.12)
Lower supervisory and technical					0.62∗	(0.12)
Semi-routine and routine					0.79	(0.14)
Unclassifiable					0.90	(0.25)
**Combined labour market status in household** (Ref. Both in work)						
Mother in work, partner not					0.90	(0.33)
Partner in work, mother not					1.05	(0.15)
Both not in work					0.96	(0.22)
Mother in work or on leave, no partner					1.04	(0.27)
**MATERNAL HEALTH BEHAVIOURS**						
**Pre-birth BMI**					0.97∗∗	(0.01)
**Breastfeed child**					1.39∗∗	(0.16)
**Drinking during pregnancy** (ref. Never)						
Less than once a month					0.98	(0.13)
1-2 times a month					1.08	(0.20)
1-2 times a week					0.85	(0.16)
3-4 times a week or more					2.64∗∗	(0.94)
**Smoking during pregnancy** (ref. Never smoked)						
Ex-smoker before pregnancy					0.81	(0.13)
Current smoker but not during pregnancy					0.85	(0.12)
Smoked during pregnancy					0.81	(0.12)
**Mother's physical exercise** (ref. Never)						
At least one a year to every few months					1.05	(0.20)
At least once a month					1.19	(0.18)
Once or twice a week					1.05	(0.13)
Every day or almost every day					1.05	(0.15)

Observations	3948		3948		3948	
Pseudo R-squared	0.073		0.109		0.180	

Notes: Dummy indicators (not shown here) are used where information on predictor variables is missing.

∗∗∗p < 0.001, ∗∗p < 0.01, ∗p < 0.05, + p < 0.10.

Mean 5 or more C-A star GCSEs is 69.8 % (SD = 45.9).

Table 3 shows that there is only a borderline significant difference in the emotional adjustment of adolescents of teenage mothers, despite the fact that such adolescents are from more disadvantaged families. Regarding physical development (Table 4), we see that adolescent offspring of teenage mothers are more likely to be overweight or obese than those whose mothers were aged 25 to 34 at the time of birth (OR = 1.40, p < 0.01). However, these differences are completely explained by selection into teenage motherhood - once this is adjusted for, the differences are no longer statistically significant. Academic attainment in high stakes exams (Table 5), measured as the probability of attaining good GCSEs (5 A∗-C), is lower among adolescents with teenage mothers (OR = 0.21, p < 0.001), and this difference is not fully explained by selection effects. When we examine the role of mediating factors relating to the early educational, emotional, material environment, and maternal health behaviours specifically relevant to children's development, we find that they completely explain the differences in educational attainment across the adolescent offspring of teenage mothers and older mothers. In particular, the home learning environment and parenting, along with income and housing, are key mediators of the relationship.

#### 3.2.2 Decomposition analyses

**Fig. 1:**
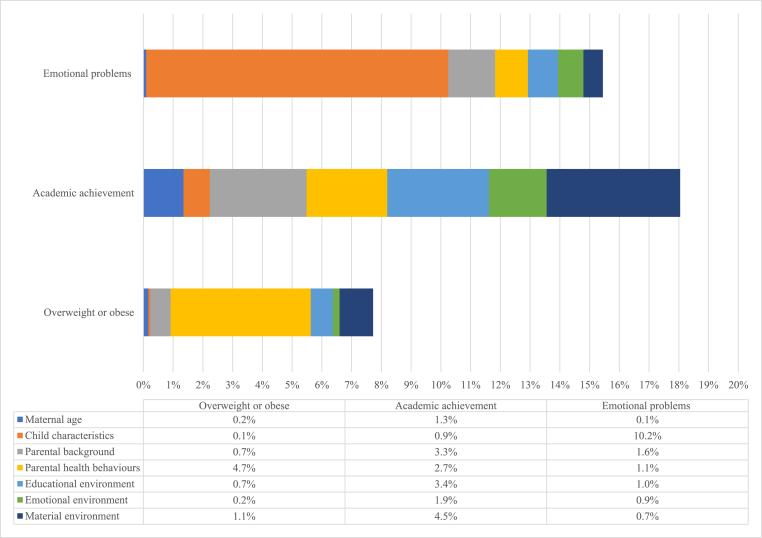
Shapley decomposition of Model 3. Note: The full regression models are shown in Table 3, Table 4, Table 5.

In order to shed light on the relative contribution of different ‘sets’ of factors in explaining children's outcomes, we decompose the proportion of the variance in each adolescent outcome explained by maternal age and each of the grouped explanatory variables. Fig. 1 shows first that in terms of the observed variation in development, the models explain between 8 % (physical development) and 18 % (academic attainment) of this.

Overall, maternal age accounts for a very low proportion of the variance (between 0.2 % and 1.3 %) compared to other variables, which vary depending on the outcome considered. The graph clearly demonstrates that multiple domains contribute to adolescent outcomes, and some more than others, depending on the specific outcome.

Considering first the determinants of socio-emotional development, shown in the top part of the figure, a key point that emerges is that the child's own characteristics (sex, birthweight) explain a dominating proportion (just over 10 %) of the total variation. This is perhaps not surprising given stark sex differences in adolescent mental health observed in multiple studies (Campbell et al., 2021). By contrast, looking at cognitive development in the middle part of the figure, there are three groups of variables that explain similar amounts of the variance: parental pre-childbearing background, the early educational environment, and the material environment. Parental health behaviours and the emotional environment explain less though are still non-negligible. This shows clearly that it is a combination of structural and behavioural factors that influences academic attainment.

Looking at physical development in the lower part of the figure, the most dominant factor to emerge is parental health behaviours, which is over half of the overall variation explained by the model. The next-biggest contributor relates to aspects of the child's material environment.

Taken together, these results highlight a range of priority areas for policy, including both structural and behavioural factors, which we return to below.

## 4 Discussion

### 4.1 Summary of findings

Our study examines the adolescent outcomes of children born to teenage mothers at the start of the millennium, a time when the adolescent birth rate in the UK stood at the highest in Europe. Using nationally representative data, it first examines the characteristics of teenage mothers around the time of birth, showing that they tended to be the most disadvantaged in terms of their backgrounds and health behaviours, and their children faced more adversity in terms of their early educational, emotional, and material environment. Later in adolescence, at age 17, their adolescent offspring experienced less favourable outcomes in two key developmental domains: they had lower academic achievement and were more likely to be overweight or obesity. No differences were observed for socio-emotional adjustment. The regression estimates showed that negative selection into teenage motherhood underpinned the observed differences in adolescent physical development. However, lower academic attainment among the adolescent offspring of teenage mothers was explained by differences between teenage and older mothers in the emotional, material and educational environment of the child.

A decomposition analysis shows that the contributions of different drivers vary considerably across the three developmental domains. For socio-emotional development, the child's own characteristics (sex, birthweight) explain a dominating proportion of the total variation, whilst the variance in academic attainment is mainly attributed to parental pre-childbearing background, the early educational and material environments. Physical development is mostly influenced by parental health behaviours, with the next-biggest contributor relating to aspects of the child's material environment.

### 4.2 Comparisons to previous studies

Whilst our approach in using rich longitudinal data to control for maternal background and accounting for the role of mediating factors is similar to others (Jaffee et al., 2001; Shaw et al., 2006), our paper adds to the literature in several ways. First, it adds new evidence for a very recently born cohort, born in 2000/2002 – just after the introduction of the ten year Teenage Pregnancy Strategy, introduced in England in 1999, marking an attempt to address the UK's historically high rates of teenage pregnancy and reduce social exclusion - and indeed provides the first evidence on long-term outcomes for the UK. Second, it studies the entire range of key developmental outcomes in late adolescence on the same sample – including academic attainment, physical health and self-reported mental health. Third, it examines the relative importance of different variables for explaining the overall variation in the outcomes of interest, using a Shorrocks-Shapley decomposition, providing further insights into the relative roles of different aspects of maternal background characteristics and behaviours in explaining development.

Findings from this study are consistent with many previous examinations, which have tended to show that children of very young mothers fare worse on a wide range of outcomes in childhood (Duncan et al., 2018; Falster et al., 2018; Sutcliffe et al., 2012), later in adolescence (Barclay & Myrskyla, 2016; Jaffee et al., 2001; Shaw et al., 2006), and into adulthood (Driscoll, 2014; Francesconi, 2008). Previous studies have also shown that the association between young maternal age and adverse offspring outcomes is largely explained by pre-existing disadvantage, ongoing resource constraints, and compromised developmental conditions such as parenting practices and other home environmental factors (Fergusson & Woodward, 1999; Mollborn & Dennis, 2012). The current study contributes further to this existing body of knowledge by examining key developmental domains in a current and UK nationally representative cohort of adolescents.

The finding of no significant difference in the emotional adjustment of adolescents of teenage mothers, despite the fact that such adolescents are from more disadvantaged families, is an interesting one. Whilst there is evidence of socio-economic inequalities in the experience of mental ill-health, the picture for adolescent mental health is more nuanced and often varies depending on the reporter. For instance, greater income-health gradients have been reported from adult reports of adolescent mental health than for adolescent self-reports (Johnston et al., 2014). Our findings are consistent with recent work using the same data which showed only a limited relationship between socio-economic position and adolescent self-reported mental health (Hazell et al., 2022).

### 4.3 Implications for policy

The decomposition analysis highlights that the contributions of different drivers vary considerably across the three developmental domains, with important implications for policy: For socio-emotional development, the child's own characteristics (sex, birthweight) explain a dominating proportion of the total variation. By contrast, three groups of variables explain similar amounts of the variance in cognition: parental pre-childbearing background, the early educational environment, and the material environment, highlighting the fact that it is a combination of structural and behavioural factors that influences academic attainment. The most dominant factor to emerge for physical development is parental health behaviours, with the next-biggest contributor relating to aspects of the child's material environment.

Whilst we focus on factors in the early childhood environment (age 9 months or 3 years) - a critical period for child development - as mediators, we recognise the strong continuity in the child's environment over time. Indeed we can evidence this in our data as shown in subsidiary analyses, so in reality the association between maternal age and outcomes in adolescence is likely to be explained by children experiencing less optimal circumstances throughout their entire childhood.

It is notable that the teenage mothers in our sample gave birth during the implementation of the UK's Teenage Pregnancy Strategy (1999–2010), meaning they had access to the enhanced supports and services introduced by that initiative. While the Strategy coincided with a sharp decline in teenage pregnancies, its long-term impact on the life chances of those who did become young mothers is uncertain - largely because comprehensive follow-up data on mother and child outcomes have been lacking (Hadley et al., 2016). Our study begins to fill this gap by examining adolescent outcomes for children born to teenage mothers in this policy context. The results indicate that most disadvantages observed among these children can be explained by pre-existing familial and socioeconomic factors rather than maternal age itself. In other words, once we account for mothers' background characteristics and the child's early environment, differences in outcomes between children of teenage mothers and those of older mothers diminish substantially. This finding suggests that the Teenage Pregnancy Strategy's supportive measures, while beneficial, did not fully overcome the deep-rooted inequalities faced by young mothers and their children. Instead, persistent issues like structural poverty and low parental education appear to be the key drivers of adolescent outcome gaps - a conclusion that aligns with prior evidence on the importance of socio-economic disadvantage in this context (Fergusson & Woodward, 1999).

Our findings carry clear policy implications. They underscore the need for initiatives that go beyond simply reducing teenage pregnancy rates to also address the entrenched disadvantages associated with teenage parenthood. In particular, tackling structural factors such as poverty and limited education is crucial for improving long-term outcomes. Future policy strategies should pair pregnancy-prevention efforts with comprehensive support for young parents. For example, improving access to education and vocational training for teenage mothers (and fathers), providing affordable childcare and flexible schooling options, and bolstering programmes that support positive parenting and maternal health behaviours would directly target the pathways influencing child development identified in our study. Likewise, strengthening economic and housing support for young families can alleviate material hardship, creating a more stable environment for children. Adopting this holistic approach - one that prevents early pregnancies where possible and robustly supports those that do occur - is likely to yield the dual benefit of further reducing teenage birth rates and improving the life chances of teenage mothers and their children. This policy direction is well-aligned with our empirical evidence and offers a path to break the cycle of disadvantage associated with adolescent parenthood.

### 4.4 Study limitations

Several limitations should be considered when interpreting our findings. First, as an observational study, causal inferences cannot be drawn. Despite extensive covariate adjustment, residual confounding remains a possibility, and unmeasured factors—such as maternal cognitive ability, paternal involvement, or neighbourhood characteristics—may influence the observed associations with maternal age. Second, attrition by age 17 may affect the generalisability of results. While inverse probability weighting was employed to mitigate bias, differential dropout, particularly along socioeconomic lines, may still influence the estimates. Third, mediating factors were measured only in early childhood. Although this is a critical developmental stage, omitting influences from later childhood and adolescence may underestimate the full impact of environmental exposures. Finally, outcome measures have inherent limitations: educational attainment was self-reported, and emotional adjustment was assessed using a brief self-report scale—both subject to potential reporting bias and limited in precision.

## 5 Conclusion

Overall, this study provides evidence on the adolescent outcomes of children born to teenage mothers in the UK around the turn of the millennium, showing that poorer academic and physical health outcomes at age 17 are largely explained by pre-existing familial, educational, and material disadvantages rather than maternal age alone. Socio-emotional outcomes did not differ significantly, underscoring the complexity of adolescent development. These findings highlight the need for policies that go beyond reducing teenage pregnancy rates to address the structural inequalities young parents face. A combined approach - preventing early pregnancies and providing sustained support for young families - offers the greatest potential to improve long-term outcomes and break cycles of disadvantage.

## Research Ethics

All procedures were performed in compliance with relevant laws and institutional guidelines and have been approved by the appropriate institutional committee(s).

Ethical approval has been obtained for all waves of the Millennium Cohort Study through the National Research Ethics Service (NRES) Research Ethics Committee (REC). The most recent ethical approval for the Age 17 Survey was obtained in October 2017, REC Ref: 17/NE/0341.

## Declaration of competing interest

The authors declare no known competing financial interests or personal relationships that could have appeared to influence the work reported in this paper.

## Data Availability

Data are available openly via the UK Data Service (UKDS)
