# Supplementary material for: Adolescent motherhood and the development of adolescent Offspring: Examining the role of selection versus early environmental factors
Source: SSM Popul Health. 2025 May 16;30:101818. doi: 10.1016/j.ssmph.2025.101818 (PMC12148460; doi:10.1016/j.ssmph.2025.101818)
Supplement: Multimedia component 1 [file mmc1.docx]

**SUPPLEMENTARY MATERIAL**

**Table S1: Measurement of explanatory variables**

| **MATERNAL BACKGROUND** | **Questionnaire items or scale** |
| --- | --- |
| **Mother’s ethnicity**  Age 9 months  Mother reported | Which of the groups on this card do you regard yourself as belonging to? (Option of 15 groups) In this study condensed to 6 groups: White, Mixed, Indian, Pakistani and Bangladeshi, Black or Black British, Other |
| **Single parent/carer**  Age 9 months  Mother reported | Derived from range of questions about who lives in the household and what their relationship is to mother |
| **Unplanned pregnancy**  Age 9 months  Mother reported | Were you planning to get  pregnant at that time or was it a surprise? |
| **Mother’s parents separated**  Age 9 months  Mother reported | Did your parents ever permanently separate or divorce? |
| **Mother in care as child**  Age 9 months  Mother reported | Before the age of 17 did you spend any time living away from both of your parents? |
| **Mother’s mother’s qualifications**  **Mother’s father’s qualifications**  Age 17 years  Mother reported | What is the highest educational qualification your mother/father has?  1 Degree level or above  2 Other Higher Education below degree level  3 A levels and equivalents  4 GCSE/O levels and equivalents  5 Another type of qualification  6 No qualification |
| **EDUCATIONAL ENVIRONMENT** |  |
| **Mother’s own qualification**  Age 9 months  Mother reported | A. Please tell me whether you have any of the qualifications on this card. If yes, please look down the list starting from the top and tell me the first one you come to that you have.  1 Higher degree  2 First degree  3 Diplomas in higher education  4 A / AS / S levels  5 O level / GCSE grades A-C  6 GCSE grades D-G  95 Other academic qualifications (incl. overseas)  96 None of these qualifications  B. Please tell me whether you have any of the qualifications on this card. If yes, please look down the list starting from the top and tell me the first one you come to that you have.  1 Professional qualifications at degree level  2 Nursing / other medical qualifications  3 NVQ / SVQ / GSVQ level 3  4 Trade apprenticeships  5 NVQ / SVQ / GSVQ level 2  6 NVQ / SVQ / GSVQ level 1  95 Other vocational qualifications (incl. overseas)  96 None of these qualifications  From these two questions the following NVQ levels of education were derived, which were used in this study:  NVQ1 (GCSE grades D-G; NVQ / SVQ / GSVQ level 1)  NVQ2 (O level / GCSE grades A-C; NVQ / SVQ / GSVQ level 2)  NVQ3 (A / AS / S levels; NVQ / SVQ / GSVQ level 3)  NVQ4 (First degree; Diplomas in higher education; Nursing / other medical qualifications (below degree level))  NVQ5 (Higher degree; Professional qualifications at degree level)  No qualifications  Overseas qual only |
| **Home learning environment**  Age 3 years  Mother reported | Measure based on frequency of five educational activities with child:  -How often do you read to [child name]?  -How often does someone at home take [child name] to the library?  -Does anyone at home ever help [child name] to learn the ABC or the alphabet?  -How often does someone at home try to teach [child name] numbers or counting?  -Does anyone at home ever teach [child name] any songs, poems or nursery rhymes? |
| **Maternal word score**  Age 14 years  Mother reported | The vocabulary scores were derived from a shortened version of the Applied Psychology Unit (APU) Vocabulary Test, a standardised test produced by the University of Edinburgh  (Closs, 1976), and used in previous studies including the 1970 British Cohort Study (BCS70) (Parsons 2014).  The version administered had 20 items, and the final score ranging from 0-20 is the sum of correct answers.  For further details see: Sullivan, A., Moulton, V., & Fitzsimons, E. (2017). The intergenerational transmission of vocabulary. *Centre for Longitudinal Studies*. |
| **Childcare**  Age 3 years  Mother reported | Questions about childcare arrange was fed forward from initial sweep and respondent was asked to confirm or change to current type. Categories used in this study were reduced to the following: Main parent or partner; Relative or friend; Childminder or nursery |
| **EMOTIONAL ENVIRONMENT** |  |
| **Maternal psychological distress**  Age 9 months  Mother reported | Mothers self-reported on the 9-item Malaise, with overall score ranging from 0-9.  Rutter, M., Tizard, J., & Whitmore, K. (1970). *Education, health and behaviour*: Longman Publishing Group. |
| **Mother-child relationship**  Age 3 years  Mother reported | Mothers completed the Pianta Child-Parent Relationship Scale, 15-item short form which measures both parent-child closeness and parent-child conflict  Driscoll, K., & Pianta, R. C. (2011). Mothers' and fathers' perceptions of conflict and closeness in parent-child relationships during early childhood. Journal of Early Childhood and Infant Psychology, (7), 1-24. |
| **Inter-parental relationship**  Age 3 years  Mother reported | Glombok-Rust Inventory of Marital State, shortened, 5-items:  Partner sensitive and aware of needs  Partner does not listen  Sometimes feel lonely even if with partner  On brink of separation  (1=Strongly agree, 2=Agree, 3=Neither agree nor disagree, 4=Disagree; 5=Strongly disagree)  How happy are you?  (1=Very happy... 2 3 4 5 6... 7=Very Unhappy)  Rust, J., Bennun, I., Crowe, M., & Golombok, S. (1986). The golombok rust inventory of marital state (GRIMS). *Sexual and Marital Therapy*, *1*(1), 55-60. |
| **Parenting type**  Age 3 years  Mother reports and interviewer observations | Parenting type was classified using 16 items related to warmth, structure, and coercive control.  Warmth:  1.It is easy to be in tune with what my child is feeling  2.I share an affectionate, warm relationship with my child  3.My child and I always seem to be struggling with each other  4.Mother's voice positive when speaking to child  5.Mother praises child spontaneously  6.Mother caresses or kisses child  Structure:  7.Which kind of family are you, one with lots of rules, or not many rules?  8.Are the rules you do have strictly enforced or not very strictly enforced?  9.Does your child go to bed at regular times?  10.Parent kept child in vision (yes, no)  11.Mother converses at least twice with child  12.Mother answers child's questions verbally  13.Mother introduces interviewer to child  Coercive control:  14.Mother scolded child more than once  15.Mother used physical restraint on child  16.Mother slapped or spanked child  Latent Class Analyses was used and which classified parents as: Authoritative, Permissive, Authoritarian, and Neglectful |
| **Inter-parental abuse**  Age 9 months  Mother reported (and partner if available) | People often use force in a relationship - grabbing, pushing, shaking, hitting, kicking etc. Has  your partner ever used force on you for any reason?  (Yes, No, Don't want to answer)  Interparental abuse identified by one or both parents responding ‘yes’. |
| **MATERIAL ENVIRONMENT** |  |
| **Household income (weekly)**  Age 9 months  Mother reported | Household income - after tax and other deductions but before housing costs - was reported by the main parent. Banded responses were used to impute continuous income, which was then equivalised to take account of household size and composition using modified OECD scales. |
| **Housing tenure**  Age 9 months  Mother reported | Do you or your partner own or rent your home or have some other arrangement? (Own outright, Mortgage, Rent Public, Rent Private, Other) |
| **Highest social class in household**  Age 9 months  Mother reported | Derived from parent and partner reported information using the the National Statistics Socio-Economic Classification (NS-SEC) (Rose & Pevalin, 2003). In this study, using the following categories:  1.Managerial and professional  2.Intermediate  3.Small employer and self-employed  4.Lower supervisory and technical  5.Semi-routine and routine  6.Unclassified  Rose, D., & Pevalin, D. J. (2003). The NS-SEC Explained. In D. Rose & D. J. Pevalin (Eds.), A Researcher's Guide to the National Statistics Socio-economic Classification (pp. 28-43). London: Sage. |
| **Combined labour market status in household**  Age 9 months  Mother reported (and partner if available) | Measure derived from a series of questions regarding employment. Categories in overall measure are:  1 Both in work  2 Mother in work, partner not  3 Partner in work, mother not  4 Both not in work  5 Mother in work or on leave, no partner  6 Mother not in work nor on leave, no partner |
| **MATERNAL HEALTH BEHAVIOURS** |  |
| **Pre-birth BMI**  Age 9 months  Mother reported | Self-reported height and weight just before becoming pregnant. BMI calculated using formula: kg/m2, where kg is a person's weight in kilograms and m2 is their height in metres squared. |
| **Breastfeed child**  Age 9 months  Mother reported | Did you ever try to breastfeed [child name]? (yes/no) |
| **Drinking alcohol during pregnancy**  Age 9 months  Mother reported | Thinking back to when you were pregnant with CM, which of these best describes how often you usually drank then?  1 Every day  2 5-6 times per week  3 3-4 times per week  4 1-2 times per week  5 1-2 times per month  6 Less than once a month  7 Never |
| **Smoking during pregnancy**  Age 9 months  Mother reported | Measure is derived based on a series of questions about current smoking and smoking during pregnancy. Categories in measure used in study are:  1 Never smoked  2 Ex smoker before pregnancy  3 Current smoker but not during pregnancy  4 Smoked during pregnancy |
| **Mother’s physical exercise**  Age 14 years  Mother reported | How often do you play sport or do any physical activity like swimming, running, football, dancing, exercise classes, going to the gym, etc.?  Response categories collapsed in current study to:  1 At least one a year to every few months  2 At least once a month  3 Once or twice a week  4 Every day or almost every day |
